# Improvement of Self-Esteem in Children with Specific Learning Disorders after Donkey-Assisted Therapy

**DOI:** 10.3390/children10030425

**Published:** 2023-02-22

**Authors:** Francesco Corallo, Lilla Bonanno, Davide Cardile, Francesca Luvarà, Silvia Giliberto, Marcella Di Cara, Simona Leonardi, Angelo Quartarone, Giuseppe Rao, Alessandra Pidalà

**Affiliations:** IRCCS Centro Neurolesi Bonino-Pulejo, S.S. 113 Via Palermo, C.da Casazza, 98124 Messina, Italy

**Keywords:** children, dyslexia, self-esteem, animal-assisted intervention, rehabilitation

## Abstract

Dyslexia is a learning disorder related to receptive language characterized by difficulties with decoding, fluent word recognition, automatic naming skills and/or reading comprehension skills. It usually leads to severe functional impairment and the permanent need for support and interventions. Since animal-assisted interventions (AAIs) have been found to improve physical, emotional, cognitive and/or social functioning in humans, the aim of this study is to demonstrate the effectiveness of onotherapy on children with SLD by improving self-esteem and school performance. Sixteen patients with a diagnosis of dyslexia were randomly assigned to two treatment groups: the first was a conventional neuropsychological group therapy without onotherapy, and the second was a neuropsychological group therapy incorporating AAIs with therapy donkeys. The neuropsychological assessment included the WISC-IV, DDE and the TMA test, which were administered before and after the treatment in both groups. The results of the experimental group show significant improvement in word reading test correctness (*p* = 0.03) and speed (*p* = 0.03), non-word reading test speed (*p* = 0.01), reading text test correctness (*p* = 0.05) and speed (*p* = 0.03), word writing test correctness (*p* = 0.01), non-word writing test correctness (*p* = 0.02), writing sentences with homophonic words correctness (*p* = 0.01), interpersonal TMA (*p* = 0.04) and the total TMA (*p* = 0.04), which were significative. On the other hand, in the control group, significant differences were found in word reading test speed (*p* = 0.01), non-word reading test speed (*p* = 0.04), reading text test speed (*p* = 0.02), writing word test correctness (*p* = 0.01), writing non-word test correctness (*p* = 0.01) and writing sentences with homophonic words (*p* = 0.01). However, in this group, we observed no significant difference in the esteem of children. Training associated with the donkeys determined improved scholastic performances as far as reading is concerned and a change in self-esteem. Therefore, we can state that AAIs for dyslexia could be a viable and effective option to enhance the rehabilitation process, increase self-esteem and improve cognitive functions and language skills recovery.

## 1. Introduction

Specific learning disorders affect 1–2.5% of the general population in the Western world, and they include a diverse group of ailments in which children with preserved intellectual capacities have problems with processing information or generating output. Learning difficulties often lead to alterations in neurocognitive processes that can manifest themselves as a deficient ability to read, speak, write, organize information, solve mathematical problems, spell, listen or concentrate [1].

Approximately 80% of people affected by learning disabilities have dyslexia, which is the most frequent learning disability [2,3,4,5,6,7]. Their etiologies are multifactorial and reflect both genetic influences [3,4] and dysfunctions in the verbal systems.

Learning Disorders (SLDs) occur in an almost constant association with other disorders (comorbidities); this determines the marked heterogeneity of the profiles and the expressiveness with which SLDs occur, and it has significant repercussions on diagnostic investigations.

Reading disability, or dyslexia, is a learning disorder based on receptive language characterized by difficulties with decoding, fluent word recognition, automatic naming skills and/or reading comprehension skills [8,9]. It generally involves severe functional impairment and the permanent need for support and interventions. Early rehabilitation was found to be a remedy in many children with dyslexia [9,10]. Learning disorders have generated several vision-based diagnostic methods and therapeutic procedures [11], sometimes without scientific support [1].

Rapid progress is being made in understanding the etiopathogenesis of many learning disorder syndromes [12,13,14], but aspects such as quality of life and the impact of the disease on families of people with learning difficulties need further investigation [15]. A very important aspect that should not be underestimated is the psychological state of the child for the situation they are living in, and this can have serious repercussions both on their work at school and their personal lives. Often the greatest difficulty is given by the onset of low self-esteem in subjects with learning disabilities.

Dyslexia is a disability that evolves over time with results that are not easily predictable. Each dyslexic child can present with a different evolution, which depends on the multiple concomitant factors that can improve or worsen the evolution.

Recent studies have examined some factors that can improve or worsen reading performance. Among these factors, Jakovljevic et al. found that gender [16] and color modifications [17] in text background and overlay influence their reading, with turquoise background/overlays and yellow backgrounds improving their performance. Moreover, some authors [18] have described the emotional and motivational aspects that characterize a child with an SLD, stating that a child with an SLD often presents a more negative self-concept, lower self-esteem, is more anxious and feels less emotional support.

The construct of well-being is very complex, and various factors intervene, such as physical and psychological health, the quality of family and friends relationships, the sense of self-efficacy and self-esteem and emotional experiences. It has been seen that among children and adolescents, a good level of self-esteem is an indication of a healthy lifestyle [19]. Some data in the literature [20] demonstrate that self-esteem in children with neuropsychiatric problems, including learning disabilities, must be assessed carefully, especially in the female gender, and there is a need for measures to prevent a trajectory toward psychopathology. Other studies have shown that therapy involving animals with people affected by intellectual impairments reports positive improvement for all psychosocial outcomes [21].

Therapies using animals to improve cognitive, emotional, physical and social functioning are called animal-assisted interventions (AAIs) [22]. The rehabilitation of children with disabilities can include the inclusion of animal-assisted therapy, as it is positively accepted by both children and families [23]. This type of therapy is used with children with different diagnoses: ADHD [24], communication disorders [25,26], Down’s Syndrome [27], ASD [28,29,30] and Cerebral Palsy [31,32]. Although the results show positive effects in relation to the treatment of children with autism, the diversity of scales used to measure the outcomes makes it occasionally difficult to establish its efficacy [33]. Moreover, these programs are used by several education and health professionals, and the results may vary depending on the participants and the animal used in the therapy [34]. Recently, some studies have shown the efficacy of equine-assisted therapy in improving the main symptoms of children with attention deficit hyperactivity disorder [35,36]. Many studies have shown that equine therapy is effective in improving self-esteem in children [37,38,39,40]; however, no studies have highlighted the effects of onotherapy on self-esteem and, consequently, on academic performance in children with learning disabilities.

Donkey-assisted therapy for patients with SLDs could represent a valid treatment strategy as part of a multimodal therapy for children with SLDs and not just a playful-recreational activity and could lead to improved self-esteem in children with SLDs. The donkey should act as a facilitator of communication between the caregiver and the patient. Children benefit greatly from contact with the animal because of its sensitivity and the natural empathy that is established between them [41].

Moreover, during onotherapy sessions, children could improve self-regulation [42] and self-care [43] and learn new skills that can be generalized to everyday life by promoting their autonomy, openness to others and self-esteem. Our study was aimed at demonstrating the effects of onotherapy on reading, writing and self-esteem in children with dyslexia. This constitutes an innovative aspect since there are currently no studies in the literature showing the effect of onotherapy on self-esteem and school performance in children with SLDs.

## 2. Materials and Methods

### 2.1. Study Design and Population

This study was conducted according to the Declaration of Helsinki. Our Institutional Ethics Committee approved the study protocol on 24 February 2021. Parents provided informed consent in both verbal and written forms.

Sixteen patients with a diagnosis of dyslexia (8 males and 8 females, a range of 7–12 years, with a median age of 9 years) with a range of 3–6 years of education that had been admitted to the Scientific Research and Care Institute “IRCCS Centro Neurolesi Bonino Pulejo” of Messina, were enrolled in this study. All of the participants were assigned randomly to one of two treatment groups: (a) a conventional neuropsychological group therapy without onotherapy (Control Group: CG n = 8); (b) a neuropsychological group therapy incorporating an AAI with therapy donkeys (Experimental Group: EG n = 8). Randomization minimizes the selection effect, and the two comparison groups allow us to determine the possible effects of the treatment compared to the group without treatment (control), while the other variables were kept constant. The inclusion criteria for this study were as follows: (1) a diagnosis of dyslexia according to the Diagnostic Statistical Manual, Fifth Edition (DSM-5); (2) aged between 8 and 11 years; (3) Q.I. normal (WISC score less than 80); and (4) the presence of low self-esteem (TMA score less than 86). The exclusion criteria were as follows: (1) the presence of other neuropsychiatric pathologies; (2) the presence of comorbidities; and (3) the presence of family conflicts that can affect self-esteem.

### 2.2. Outcome Measures

The neuropsychological assessment in this study included the Wechsler Intelligence Scale for Children-IV (WISC-IV) [43], the Italian Battery for Evaluation of Dyslexia and Dysorthography (DDE) [44] and the TMA test (Multidimensional Self-Esteem Test) for the evaluation of self-esteem age of development in its many dimensions.

The WISC-IV is a standardized clinical tool used for the cognitive assessment of children and young people aged between 6 and 16 years. The four cognitive areas assessed by WISC IV correspond to specific indices, as follows: the Verbal Comprehension Index (VCI), the Visual–Perceptual Reasoning Index (PRI), the Working Memory Index (WML) and the Processing Speed Index (PSI). Moreover, WISC-IV allows, from the sum of these indices, the elaboration of three composite ones, as follows: one index for Global Intellectual Quotient (IQ), one index for General Ability (IAG) and at least one for Cognitive Competence (ICC). WISC-IV is mainly used to support diagnostic hypotheses in the assessment of children with specific learning disorders (SLDs). In Italy, in fact, to diagnose an SLD, the subject must have an IQ of at least 85 and a significant discrepancy between their scholastic performance and their IQ [45].

The DDE is a battery used in the assessment of the level of competence acquired in writing and reading, and it is very useful for monitoring progress during and after treatment. The DDE is divided into 8 tests. Of these tests, 5 aim to assess and analyze the reading process, requiring the subject to name graphemes, read words and non-words, understand sentences with homophones and correct homophones. On the other hand, the remaining 3 tests of the battery aim to assess and analyze the reading process by asking the subject to write words and non-words under dictation and write sentences with homophonic words under dictation. This battery has been recognized by the Italian Dyslexia Association as part of the basic diagnostic protocol for the evaluation of disorders in terms of writing, reading and calculation. It also makes it possible to compare pre- and post-treatment performance and promote communication between rehabilitation centers and operators.

The TMA [46] assesses self-esteem by deepening six areas, as follows: the interpersonal area (evaluating a subject’s perception of their social relationships with peers and adults), the school area (a subject’s sense of success or failure in the classroom), the emotional area (delving into the emotional sphere and assessing the ability to control negative emotions), the family area (a subject’s perceptions of family relationships and the degree to how important and loved they feel, etc.), the body area (a subject’s perception of their appearance, physical and sporting skills, etc.) and the area of mastery over the environment (a subject’s feeling of being able to dominate the events of one’s life, etc.).

### 2.3. Procedures

One group (EG) underwent bi-weekly individual neuropsychological training associated with donkey-assisted therapy training once a week, and a control group (CG) underwent traditional individual neuropsychological training. Children with SLDs were recruited and diagnosed at our Child Neuropsychiatry clinic with the following battery: detailed medical history, neuropsychiatric visit and WISC-IV for an intelligence assessment. School performance was assessed using the DDE battery for the assessment of reading, writing and arithmetic. The level of self-esteem was assessed through the Italian version of the Multidimensional Self-Esteem Test—TMA. The training lasted for 6 months, at the end of which the children were re-checked again with the administration of school tests and TMA.

### 2.4. Donkey-Assisted Training

Each training session lasted 45 min and was divided into 5 phases of 10 min each, unlike the last one, which lasted 5 min. The first phase of donkey-assisted training consists of interaction with the donkey and an introduction to the environment in which they live. This was followed by a phase of grooming, cleaning and physical contact to teach the children basic safety rules, animal anatomy and etiology and animal management issues. The third phase involved the children conducting the donkey along the path used for exercise, and the fourth phase was dedicated to saddling, riding and leading the children on the donkey in the field while invited to perform specific exercises. From these two phases, the children could learn basic aspects of riding (such as guiding the donkey around objects, mounting, dismounting, positioning, walking and trotting). This allows the children to increase motor abilities and coordination, self-esteem and sensor perception. During the last phase, the children were dismounted and encouraged to socialize and relate to the animal for greetings (saying “goodbye” and hugging the donkey).

## 3. Statistical Analysis

With regard to statistical analysis, nonparametric analysis was performed because the results of the Shapiro normality test highlighted a non-normal distribution of most of the target variables. The numerical data are presented as the median and the first-third quartile as a non-normal distribution. The Wilcoxon signed-rank test and the Mann–Whitney U test were utilized for intra and inter-group analysis, respectively. An interaction effect analysis (improved time) was performed by submitting the T1–T0 differences in the variables scores to correlation and regression analyses. Spearman correlation was used to evaluate whether there was a relationship between the DDE battery and the TMA sub-test. The analyses were performed using an open source R3.0 software package. The confidence level was set to 95% with a 5% alpha error. Statistical significance was set at *p* < 0.05.

## 4. Results

The Wilcoxon signed-rank test showed a significant difference in the experimental group between T0 and T1 (Table 1).

**Figure 1:**
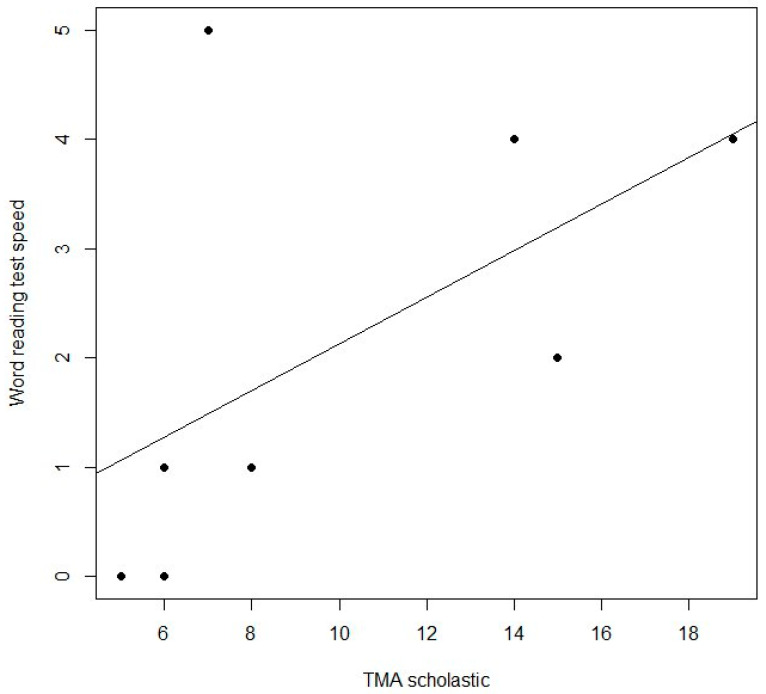
Correlation between scholastic TMA and word reading test speed in experimental group. The X-axis and Y-axis represent the differences in T1-T0 scores on the TMA scholastic and word reading tests, respectively.

In particular, word reading test correctness (*p* = 0.03) and speed (*p* = 0.03), non-word reading test speed (*p* = 0.01), reading text test correctness (*p* = 0.05) and speed (*p* = 0.03), word writing test correctness (*p* = 0.01), non-word writing test correctness (*p* = 0.02), writing sentences with homophonic words correctness (*p* = 0.01), interpersonal TMA (*p* = 0.04) and total TMA (*p* = 0.04) were significant. Moreover, a trend was present between non-word reading test correctness (*p* = 0.07), emotional TMA (*p* = 0.08) and scholastic TMA (*p* = 0.08) between T0 and T1. In the control group, we found significant differences in word reading test speed (*p* = 0.01), non-word reading test speed (*p* = 0.04), reading text test speed (*p* = 0.02), writing word test correctness (*p* = 0.01), writing non-word test correctness (*p* = 0.01) and writing sentences with homophonic words (*p* = 0.01). In this group, we have no heightened significant difference between T0 and T1 in the TMA sub-test. In the inter-group, we found a significant difference in the word writing test (*p* = 0.02) at T1 (Table 1). Spearman correlation showed a trend between word reading test speed and scholastic TMA (*p* = 0.07) in the experimental group (Figure 1).

People with specific learning disorders may have lower levels of self-esteem and present more difficulties in terms of emotional and behavioral fields than those without dyslexia. However, the nature of the relationship between self-esteem and psychopathology remains unknown [47]. This study reports results from a small sample of patients with dyslexia and low self-esteem involved in a 6-month AAI intervention compared to an activity control group that had no interaction with a donkey. The results show significant post-intervention improvements in the AAI group compared to the group control. In fact, the results obtained confirm that onotherapy associated with traditional therapy can help improve school performance and self-esteem in children with dyslexia. We considered only children between the ages of 8 and 11 to avoid the presence of subjects who might have adolescent problems that could result in lowered self-esteem. We also excluded children with socio-familial problems, considering that self-esteem can be damaged by situations other than dyslexia. 

There were significant improvements in both the control group and the experimental group. While in the control group, the improvements concern only word reading test speed, non-word reading test speed, reading passage speed test, writing word test correctness, writing non-word test correctness and writing homophone word test correctness, but there were no improvements in the esteem of the children, in the group who underwent onotherapy associated with traditional training, in addition to the improvements already recorded in the control group, there is also an improvement in the correctness of reading the passage but above all in both global and interpersonal self-esteem. It is evident that the positive change in dyslexia is the result of the traditional cognitive training of reinforcement of the deficient areas, but the training associated with the donkey determined improved scholastic performances as far as reading is concerned and a positive change in the self-esteem values, which were not present in the control group. There is a lack of high-quality evidence on which to base any decision about the use of animal-assisted therapy for dyslexic children. Previous studies have demonstrated that children with reading difficulties presented decreased emotional and executive function abilities [48]. There is a consistent association between academic self-esteem, emotional symptoms and internalizing difficulties in dyslexia. There are multiple treatments and interventions that ameliorate dyslexia symptoms in children [49]. It is extremely important to evaluate the etiology of the disturbances to adequately plan the intervention. In fact, any intervention must also take into consideration any comorbidities to be as complete as possible [50].

The various intervention methods used to improve the literacy, cognitive function and reading ability of children with dyslexia include the use of colored backgrounds or layouts [16,17], multisensory and multimedia methods [51] and virtual reality [52,53]. The combination of cognitive functions, linguistic literacy deficits and self-esteem interventions is not provided in any study in the literature. Children with dyslexia present with deficits in higher-order processing or executive control processes. They also have peculiar eye movement tendencies [54], deficits in visual attention span, processing speed, verbal working memory [55,56] and difficulties with memorizing. Animal-assisted interventions represent an innovative rehabilitation approach that can improve the social, emotional and physical aspects of several diseases. Few data are available regarding donkey therapy. The results support that the use of donkey therapy for children with SLDs can improve self-esteem and obtain improved learning performance.

The outcomes of this study suggest that there is an important function in the child–donkey interaction that can affect positive changes in terms of self-esteem and learning.

Our results generate hypotheses regarding the role of the child–donkey interaction requiring further investigation. One hypothesis is that working together with the donkey involves the stimulation of nonverbal joint attention, shared attention, executive attention and memory that serve as a platform for improving linguistic abilities. Additionally, motor training with the donkey also has positive effects on several cognitive domains, including attention, memory, processing speed and inhibition beyond mood and mobility. Another hypothesis is that the child’s experience with the equine (i.e., the warmth of the donkey’s body) creates a calming context, which could have a calming effect on children with dyslexia and, consequently, improve their performance in school tests due to an increased ability to concentrate. In addition, motor training with the donkey, featured in our protocol, could bring about positive effects on several cognitive domains, including memory, attention, inhibition and processing speed, in addition to acting on mood and mobility. All of this would, in our opinion, promote the improvement of self-esteem in the experimental group, as evidenced by the results obtained, reporting an increase in the total score on the total TMA score and in the interpersonal TMA score. Self-esteem could be improved thanks to the human–donkey interaction defined as “total grooming”, as it is linked to the idea of parental care, where the patient is rocked and pampered by the parents [57]. The relationship and the emotional attachments that children establish with a donkey has a positive influence on several fronts. Social functioning seems to benefit from the children–animal contact as well as executive abilities [58]. Specific reference is made to animal contact because, occasionally, this in itself has been shown to improve the individual’s behavior, executive function, sustained attention, working memory and probably even emotional control [59]. Another aspect of the equines that can elicit a positive change in patients is its movement. In fact, the animal, when moving, stimulates several systems simultaneously, such as the limbic, sensory, skeletal, muscular and vestibular systems [60]. Moreover, a recent meta-analysis [61] specifies that Equine-Assisted Therapies reduce reaction time in problem-solving situations and maladaptive behaviors while improving socialization and engagement in ASD patients.

A diagnostic category that is often not referred to when discussing equine-assisted therapy is mental illness. A study conducted by Punzo et al. found that children and adolescents suffering from mental illness find relief from everyday anxieties and fears and improve their self-esteem thanks to the friendship established with the animal, strengthening their self-confidence [62]. Similar results were found by Alfonso et al. [63], who noted in their study that an equine-assisted intervention was highly effective in reducing symptoms and signs of social anxiety in young women aged 18–29. These results might make one consider the idea of combining equine-assisted therapy with psychiatric treatments to decrease the prescription of psychiatric drugs. Recent studies have investigated the effects of equine-assisted therapy in fields of application that are still partially unexplored, namely in the treatment of obesity, substance use disorders, post-traumatic stress disorder (PTSD) and work-related stress. An exploratory case study of Schroeder et al. [64] shows that equine therapy is perceived as acceptable and enjoyable by the participants, who benefit from it as it increases their physical activity and their self-efficacy for physical exercise. Similar studies had already been carried out on at-risk adolescents and their body image, self-control, confidence and satisfaction [65], but not in patients with obesity. The intervention was also found to have a positive effect on the treatment of patients suffering from substance use disorders [66]. The latter perceived the contact with the animal as a break from traditional treatments, and positive results were recorded both in the retention and completion of treatment. As regards post-traumatic stress disorder, a study conducted on veterans expressed the potential of AAI in reducing PTSD symptoms [67]. Finally, the treatment also seems to find use in work environments. Healthcare workers are among the categories most affected by stress and burnout, and this is why a very recent study investigated the safety, feasibility and perception of participants with a resiliency intervention that included AAIs [68]. The results showed that the participants found it enjoyable, and there was a significant increase in psychological flexibility. While conventional wisdom has always affirmed the value of animals in promoting human well-being, only recently has their therapeutic role in medicine become the focus of dedicated research [22]. We believe that a donkey AAI has led to better outcomes through specific animal training that encourages active communication, enhances engagement, brings enjoyment and motivation and increases treatment compliance. The latter strongly relates to another fundamental aspect that is often put in the background or even neglected: satisfaction. In their review, Siewertsen et al. [69] have shown that pet therapy (with both dogs and equines) has a high satisfaction index not only among participants but also and especially among their families. When satisfaction is high, it tends to increase the commitment and frequency with which parents are willing to place their resources into treatment, thus limiting the risk of drop-out. However, although the benefits of equine-assisted activities and therapies have been amply demonstrated by many studies, the implementation of this type of treatment remains limited due to the lack of standardized treatments and systematic theory-based knowledge. This is the first study evaluating the effects of an AAI in children with dyslexia and low self-esteem. Our study also showed a trend of improvement after the AAI in terms of emotional and school TMA. These data could be confirmed with the significance of the results by increasing the sample size. Effectively, the main limitations of this study consist of the relatively small sample size, which limits the generalizability of our data and the lack of an adequate long-term follow-up. Therefore, we can state that the use of AAIs for dyslexia could be feasible and effective as it allows us to enhance the rehabilitation process, improving self-esteem and increasing the recovery of language skills as well as cognitive functions.

## 5. Conclusions

In conclusion, this study demonstrates that an AAI using donkeys in a rehabilitation program could be a motivational and effective tool for enhancing language skills, favoring cognitive functions, promoting psychological well-being and improving self-esteem. Further larger sample studies with long-term follow-up periods are needed to confirm the effect of this interesting approach. The conclusions are preliminary, and the limitations of the study include the small number of subjects and a lack of a longer follow-up, and that it is not randomized. The value of the current small study only points in one direction, increasing self-esteem in children with dyslexia, and no firm conclusions can be drawn yet.

## Figures and Tables

**Table 1:** Inter and Intra group analysis.

			Patient	Control	*p*
			Median (Min–Max)	Median (Min–Max)	
Word reading test	CORRECTNESS PERCENTILE	T0	7 (6–10)	9 (2–10)	0.62
		T1	10 (10–10)	10 (10–10)	NA
		*p*	0.03 *	0.1	
	SPEED PERCENTILE	T0	6.5 (2–8)	5.5 (4–9)	1
		T1	8 (6–10)	8.5 (8–10)	0.19
		*p*	0.03 *	0.01 *	
Non-word reading test	CORRECTNESS PERCENTILE	T0	8 (1–10)	9 (7–10)	0.31
		T1	10 (8–10)	10 (8–10)	0.94
		*p*	0.07	0.18	
	SPEED PERCENTILE	T0	7.5 (3–8)	8 (7–10)	0.15
		T1	9 (7–10)	8.5 (8–10)	0.91
		*p*	0.01 *	0.04 *	
Reading text test	CORRECTNESS PERCENTILE	T0	9 (6–10)	9.5 (8–10)	0.74
		T1	10 (10–10)	10 (9–10)	0.39
		*p*	0.05 *	0.09	
	SPEED PERCENTILE	T0	5 (4–7)	6 (4–8)	0.4
		T1	8 (5–9)	8 (8–9)	0.2
		*p*	0.03 *	0.02 *	
Word writing test	CORRECTNESS PERCENTILE	T0	1 (1–4)	1.5 (1–7)	0.81
		T1	7.5 (2–10)	9.5 (8–10)	0.02 *
		*p*	0.01 *	0.01 *	
Non-word writing test	CORRECTNESS PERCENTILE	T0	5.5 (1–10)	5.5 (1–9)	0.91
		T1	10 (9–10)	10 (9–10)	1
		*p*	0.02 *	0.01 *	
Writing sentences with homophonic words	CORRECTNESS PERCENTILE	T0	1 (1–7)	1.5 (1–7)	0.86
		T1	9 (3–10)	9 (9–10)	0.61
		*p*	0.01 *	0.01 *	
TMA	INTERPERSONAL P.LE	T0	19.5 (8–91)	33.3 (1–40)	1
		T1	50 (20–95)	36 (1–4)	1
		*p*	0.04 *	0.37	
	COMPETENCE P.LE	T0	24 (1–79)	22.5 (1–48)	1
		T1	24 (1–75)	22.5 (1–48)	1
		*p*	0.23	NA	
	EMOTIONAL P.LE	T0	25 (8–86)	37.5 (8–50)	1
		T1	50 (10–90)	38.5 (8–60)	1
		*p*	0.08	0.37	
	SCOLASTIC P.LE	T0	19 (1–87)	10.5 (1–48)	1
		T1	27.5 (20–95)	17 (1–54)	1
		*p*	0.08	0.18	
	FAMILIAR P.LE	T0	12.5 (1–80)	6 (1–51)	1
		T1	17.5 (1–80)	6 (1–51)	1
		*p*	0.27	NA	
	CORPOREAL P.LE	T0	31 (5–69)	32.5 (3–91)	1
		T1	31 (6–69)	32.5 (3–91)	1
		*p*	0.55	NA	
	TOT P.LE	T0	13.5 (1–79)	26 (1–52)	1
		T1	24 (20–82)	26 (1–52)	1
		*p*	0.04 *	1	

* *p* < 0.05.

## Data Availability

Not applicable.
